# Induction of neural differentiation in rat C6 glioma cells with taxol

**DOI:** 10.1002/brb3.414

**Published:** 2015-10-26

**Authors:** Chuan‐Chuan Chao, Daphne Kan, Ta‐Hsuan Lo, Kuo‐Shyan Lu, Chung‐Liang Chien

**Affiliations:** ^1^ Department of Anatomy and Cell BiologyCollege of MedicineNational Taiwan UniversityTaipeiTaiwan; ^2^ Center of Genomic MedicineNational Taiwan UniversityTaipeiTaiwan

**Keywords:** Glioma, neural differentiation, taxol

## Abstract

**Background:**

Glioblastoma is a common and aggressive type of primary brain tumor. Several anticancer drugs affect GBM (glioblastoma multiforme) cells on cell growth and morphology. Taxol is one of the widely used antineoplastic drugs against many types of solid tumors, such as breast, ovarian, and prostate cancers. However, the effect of taxol on GBM cells remains unclear and requires further investigation.

**Methods:**

Survival rate of C6 glioma cells under different taxol concentrations was quantified. To clarify the differentiation patterns of rat C6 glioma cells under taxol challenge, survived glioma cells were characterized by immunocytochemical, molecular biological, and cell biological approaches.

**Results:**

After taxol treatment, not only cell death but also morphological changes, including cell elongation, cellular processes thinning, irregular shapes, and fragmented nucleation or micronuclei, occurred in the survived C6 cells. Neural differentiation markers NFL (for neurons), *β* III‐tubulin (for neurons), GFAP (for astrocytes), and CNPase (for oligodendrocytes) were detected in the taxol‐treated C6 cells. Quantitative analysis suggested a significant increase in the percentage of neural differentiated cells. The results exhibited that taxol may trigger neural differentiation in C6 glioma cells. Increased expression of neural differentiation markers in C6 cells after taxol treatment suggest that some anticancer drugs could be applied to elimination of the malignant cancer cells as well as changing proliferation and differentiation status of tumor cells.

## Introduction

Glioblastoma is the most common and aggressive type of primary brain tumor in humans. Although it can be treated with surgical removal, radiation, and chemotherapy, the prognosis and outcome is still unsatisfying. When treating GBM (glioblastoma multiforme) cells with various anticancer drugs, not only cell death but also morphological changes have been observed. For example, with a combination of radiation, rapamycin triggers neural differentiation of glioma‐initiating cells isolated from human GBMs (Zhuang et al. 2011). Taxol is an anticancer drug that has been used in the treatments of many solid tumors, such as breast, ovarian, and prostate cancers (Ranganathan et al. 1996, 1998; Kavallaris et al. 1997). In vitro, taxol enhances the polymerization of tubulin into stable microtubules by interacting directly with microtubules and stabilizing them against cold or calcium‐induced depolymerization (Horwitz 1994). In ovarian cancer cell line, skov3, taxol inhibits the tumor growth by inducing morphological change (Jia et al. 2012). Taxol can also promote myeloid‐derived suppressor cells differentiate into dendritic cells in vitro (Michels et al. 2012). Taxol‐administrated U87MG xenografts in nude mice show increased expression of GFAP (glial fibrillary acidic protein), which indicates astrocytic differentiation promoted in glioma cells (Karmakar et al. 2008). However, the mechanism and effects of taxol‐induced GBM cell neural differentiation are still unclear.

Rat C6 glioma cell line was established by exposure of outbred Wistar rat astrocytes to *N, N'*‐nitroso‐methylurea (Benda et al. 1968; Schmidek et al. 1971). When injecting C6 cells into new born rats, tumor that consistent with GBM pathologically could be induced (Auer et al. 1981). Thus, C6 glioma cell line can serve as an in vivo experimental astrocytoma model, which is also useful in investigating the mechanism of tumor growth, angiogenesis, invasion, and designing anticancer therapies (San‐Galli et al. 1989; Bernstein et al. 1990, 1991; Nagano et al. 1993; Abramovitch et al. 1995; Chicoine and Silbergeld 1995). C6 glioma cells have also been applied for studying neural differentiation, for example, astrocytic differentiation of C6 glioma cells is induced by administration of dbcAMP (dibutyryl cAMP) (Messens and Slegers 1992; Hu et al. 2008). Induced oligodendrocytic differentiation has been also identified by the expression of glutamate transporter excitatory amino acid carrier 1 (EAAC1) in C6 cells after retinoic acid treatment (Bianchi et al. 2008).

In this study, we present the effects of taxol challenge on rat C6 glioma cells in the aspects of cellular morphology and differentiation. Characterizations of neural molecular markers including *β*III‐tubulin (for neurons), GFAP (for astrocytes), and CNPase (2′, 3′‐cyclic nucleotide 3′‐phosphodiesterase for oligodendrocytes) in the taxol‐treated cells revealed that taxol might trigger neural differentiation. The differentiation pattern induced by taxol was compared with that caused by dbcAMP. We also performed quantitative analysis to examine various neural cell‐type‐specific molecular markers in the differentiated cells after taxol treatment. Based on the fact that neural differentiation could be promoted in taxol‐treated glioma cells, a potential therapeutic application of anticancer drugs on the differentiation of tumor cells was suggested.

## Materials and Methods

### Cell culture

Rat C6 glioma cell line was purchased from the BCRC (Bioresource Collection and Research Center; Taiwan). Cells have been cultured in the serum‐containing medium composed of DMEM (Dubecco's modified Eagle's medium) with high glucose (Life Technologies, Carlsbad, CA), 10% fetal bovine serum (FBS, Life Technologies) and penicillin‐streptomycin (Life Technologies). Cells were incubated at 37°C with 95% air, 5% CO_2_, and 100% humidity.

### Drug treatment

C6 cells were plated at a density of 1 × 10^5^ cells/6‐cm dish. After 24 h incubation, cells were exposed to medium containing dbcAMP (100 nmol/L, Sigma‐Aldrich, St. Louis, MO) or different concentrations of taxol (Sigma‐Aldrich) for 48 h. Cells were collected for western blot analysis and immunocytochemistry after the treatments.

### Cell viability assay (MTT assay)

C6 cells were plated at a density of 1000 cells/well in 96‐well tissue culture plates (Corning Inc., Corning, NY). Cell proliferation and viability were assessed with the MTT ([3‐(4, 5‐dimethylthiazol‐2‐yl)‐2, 5‐diphenyl‐tetrazolium bromide], Sigma‐Aldrich) assay. At the end of treatment, fresh medium with 0.5 mg/mL of MTT was added for a 4‐h incubation. Blue formazan crystals formed in the cells were dissolved in 100 *μ*L DMSO, and the absorbance was measured at 570 nm using a Multiskan FC ELISA reader (Thermo Fisher Scientific, Waltham, MA).

### Immunofluorescence staining

Cells used for immunofluorescence studies were cultured on poly‐D‐lysine‐coated (Sigma‐Aldrich) coverslips, fixed with 4% paraformaldehyde (Sigma‐Aldrich) in PBS (pH 7.4) for 10 min, washed three times with PBS and incubated with blocking buffer containing 10% normal goat serum (Life Technologies) in PBS‐0.1% triton X‐100 for 30 min at room temperature. After removing the blocking buffer, cells were incubated overnight with primary antibodies against Nestin (1:1000, mouse monoclonal; BD Biosciences, San Diego, CA; Cat# 611658, RRID: AB_399176), *β*III‐tubulin (1:1000, rabbit polyclonal IgG; Sigma‐Aldrich‐Aldrich Cat# T2200, RRID: AB_262133), GFAP (1:200, mouse monoclonal; Sigma‐Aldrich‐Aldrich Cat# G3893, RRID: AB_477010), or CNPase (1:200, mouse monoclonal IgG; Millipore, Billerica, MA; Cat# MAB326, RRID: AB_2082608) at 4°C. After washing three times with PBS at room temperature, cells were incubated with fluorophore‐conjugated secondary antibodies (Sigma‐Aldrich) that recognized primary antibodies. Hoechst 33342 (Sigma‐Aldrich) was used to label cell nuclei. After washing with PBS three times at room temperature, cells were mounted in fluoromount G (Electron Microscopy Science, EMS, Hatfield, PA). Cells were visualized with a Leica TCS SP5 confocal microscope (Leica Microsystems GmbH, Wetzlar, Germany).

### Immunocytochemistry for the quantification of differentiation

Cells were fixed with 4% paraformaldehyde (Sigma‐Aldrich) in PBS (pH 7.4) for 10 min, washed three times by PBS, and then treated with 0.3% H_2_O_2_/PBS for 10 min at room temperature to remove endogenous peroxidase followed by incubation with blocking buffer containing 10% normal goat serum (Life Technologies) and 0.1% triton X‐100 (Sigma‐Aldrich) in PBS for 30 min at room temperature. After removing the blocking buffer, cells were incubated overnight with primary antibodies against *β*III‐tubulin (1:1000, rabbit polyclonal IgG), GFAP (1:200, mouse monoclonal), and CNPase (1:200, mouse monoclonal IgG) at 4°C. After washing the cells three times with PBS at room temperature, cells were incubated with biotin‐conjugated secondary antibodies (Sigma‐Aldrich) for 1 h to recognize primary antibodies. Cells were rinsed three times with PBS at room temperature then incubated in ABC (avidin‐biotin complex, Vector Elite kit, Vector Laboratories, Burlingame, CA) for 1 h. Following three times washes, cells were exposed to the substrate, 3, 3′‐diaminobenzidine tetrachloride (DAB, Vector Laboratories) in PBS buffer and 0.003% H_2_O_2_. The brown‐immunoreactive products appeared. The cells were then washed in water and counterstained with hematoxylin, dehydrated in absolute alcohol, cleaned in xylene and mounted with Permount. Positive staining was examined and photographed under a Leica microscope, and three randomly selected fields (800 *μ*m × 600 *μ*m) from each immunostaining were captured for quantification. Data were represented as mean ± SEM for three experiments in each group. *P* values below 0.01 were considered statistically significant by using unpaired Student's *t*‐test.

### Real‐time PCR analysis

Cells were collected and processed for total RNA extraction by Trizol (Life Technologies) according to the manufacturer's instructions. One *μ*g total RNA was reverse‐transcribed to first‐strand cDNA by oligo‐dT primer provided in ThermoScript^™^ RT‐PCR System (Life Technologies). The primers for the marker genes are shown in Table 1. Real‐time PCR was carried out on cDNA using Brilliant II SYBR Green QPCR Master Mix Kits with low Rox (Agilent Technologies, Inc., Santa Clara, CA) in MX3000P PCR cycler (Agilent Technologies, Inc.). All reactions were performed in a 25‐*μ*L volume using the following PCR program: initial denaturation at 95°C for 10 min, 40 amplification cycles at 95°C for 30 sec, 60 sec at 60°C, and 60 sec at 72°C. PCR with H_2_O instead of the template was used as a negative control. Specificity was verified by melting curve analysis and agarose gel electrophoresis. The threshold cycle (Ct) values of each sample were used in the post‐PCR data analysis. *gapdh* was used as an internal control for mRNA level normalization.

**Table 1:** Primer sets

Gene	Sequence (5′‐>3′)	Size	Accession no.
*gapdh*	F: CATCAAGAAGGTGGTGAAGCAGG	206 bp	NM_017008
	R: CCACCACCCTGTTGCTGTAGCCA		
*nestin*	F: CTTAGTCTGGAGGTGGCTACATACA	423 bp	NM_012987
	R: GAGGATAGCAGAAGAACTAGGCACT		
*gfap*	F: ATTCCGCGCCTCTCCCTGTCTC	437 bp	NM_017009
	R: GCTTCATCCGCCTCCTGTCTGT		
*tubb3*	F: AAGGCATGGATGAGATGGAG	279 bp	NM_139254
	R: GTGCCCTGAAGAGCTGGTAG		
*cnp*	F: AACTGTCCATCTCTGCCCTC	194 bp	NM_012809
	R: GTCAAGGCCTGTCTGCACTG		

### Western blot analysis

For preparation of protein lysates, cells were washed by cold PBS, and then lysed in RIPA protein lysis buffer (150 mmol/L NaCl, 1% NP‐40, 0.5% deoxycholic acid, 0.1% SDS, 50 mmol/L Tris‐HCl at pH 7.5, and 5 mmol/L EDTA) containing protease inhibitor cocktails (Roche Applied Science, Mannheim, Germany). The cell debris was removed by centrifugation at 14,000 rpm for 30 min at 4°C. The protein concentration in the supernatants compared to standard BSA concentrations was determined by Bio‐Rad protein assay kit (Bio‐Rad, Hercules, CA). 30 *μ*g total proteins of cell lysates was loaded in each lane of 9% SDS‐PAGE gel. The proteins were separated after electrophoresis and subsequently transferred onto PVDF membranes (Bio‐Rad). The membranes were probed with specific antibodies against *β*III‐tubulin (1:1000, rabbit polyclonal), GFAP (1:1000, mouse monoclonal), CNPase (1:1000, mouse monoclonal), and Nestin (1:1000, mouse monoclonal). The anti‐GAPDH antibody (1:25,000, mouse monoclonal, Sigma‐Aldrich, Cat# G8795, RRID: AB_1078991) was used to probe the GAPDH internal control for equal loading of protein lysates. Immunocomplexes were formed with primary antibodies by incubating the proteins overnight at 4°C. Blots were washed and incubated with peroxidase conjugated anti‐mouse secondary antibodies (GE Healthcare, Milwaukee, WI) for 1 h. The Western blot analysis was detected with ECL western blotting system (Millipore).

## Results

### The effects of different taxol concentrations on C6 cells

**Figure 1:**
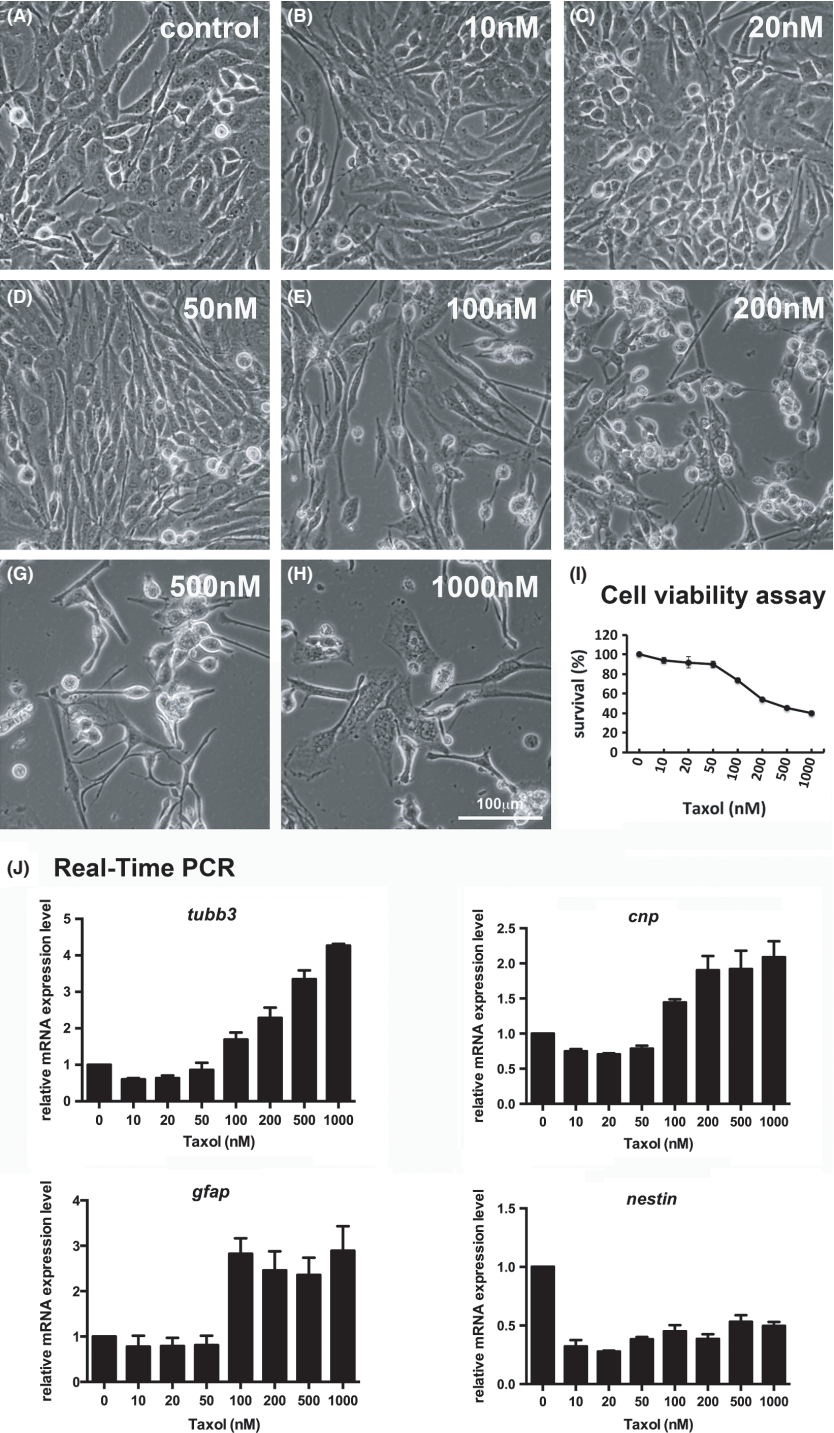
The morphological effect of C6 cells after 48 h taxol treatment. C6 cells were cultured in medium composed of Dubecco's modified Eagle's medium and (A) 10% FBS and with different concentrations of taxol, including (B)10, (C) 20, (D) 50, (E) 100, (F) 200, (G) 500, and (H) 1000 nmol/L for 48 h. The morphological images of the cells were recorded by phase contrast microscopy. Bar scale = 100 *μ*m. (I) Cell viability was measured by MTT assay after 48‐h incubation. (J) Real‐time PCR data showed that apparent increases in mRNA levels of *tubb3* (*β* III‐tubulin), *gfap,* and *cnp* when taxol concentration reached 100 nmol/L. mRNA of *nestin* was downregulated in taxol‐treated C6 cells. Data are presented as the mean ± SEM for three experiments in each group.

After taxol treatment, the cellular responses of C6 cells were recorded by phase contrast images (Fig. 1). In the untreated control group, C6 cells proliferated normally (Fig. 1A). Some of the taxol‐treated C6 cells showed morphological changes, such as became large and round. The phenomenon was especially observed in the cells exposed to 100 nmol/L and higher concentrations of taxol (Fig. 1E–H). After 48 h treatment, cell elongation, cellular processes thinning, irregular shapes, and fragmented nucleation or micronuclei were observed. The morphology turned into neural cell‐like appearances, which indicated possible neural differentiation induced by taxol challenge. MTT assay was performed to measure cell viability in the end of drug treatments. After 100 nmol/L taxol treatment, 74% cells survived in relative to the control experiment (without taxol treatment). The survival rate decreased to 40% when taxol concentration increased to 1000 nmol/L. The dosage effects of taxol challenge on C6 cell viability were shown in Figure 1I. Moreover, mRNA expression levels of neural differentiation markers, *tubb3* (*β*III‐tubulin), *gfap*,* cnp,* and *nestin* were examined by real‐time PCR (Fig. 1J). Apparent increases in mRNA levels of neural differentiation markers were observed in the experiments of 100 nmol/L and higher concentrations of taxol. The concentration of 100 nmol/L was chosen for the subsequent experiments.

### Upregulation of neural differentiation markers in taxol‐treated C6 cells

**Figure 2:**
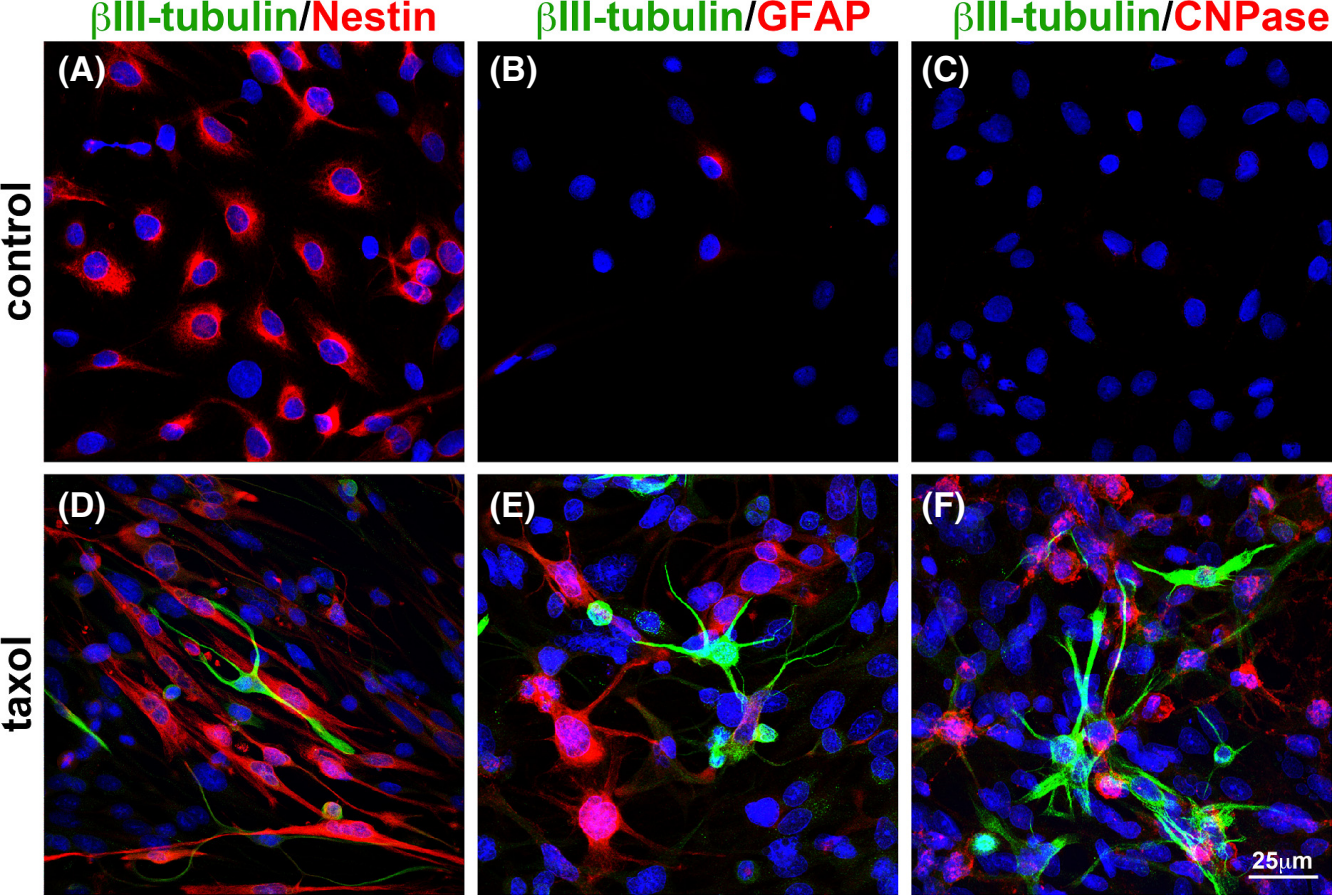
The expression of neural differentiation markers induced by taxol in C6 cells as demonstrated by immunofluorescent staining. Control (untreated) C6 cells and 100 nmol/L taxol‐treated C6 cells were analyzed by immunofluorescent staining at 48 h. In control cells, (A) the neural stem cell marker nestin was detected, (B) very few cells carrying GFAP protein could be found, and (C) immunoreactivites for *β* III‐tubulin and CNPase were difficult to detect. (D–F) The immunoreactivities for neuronal marker *β* III‐tubulin, astrocyte marker GFAP, and oligodendrocyte marker CNP, were clearly identified in the C6 cells after treated with taxol. These results indicate that expression of the neural differentiation markers were induced when the cells were challenged by taxol. Bar scale = 25 *μ*m.

**Figure 3:**
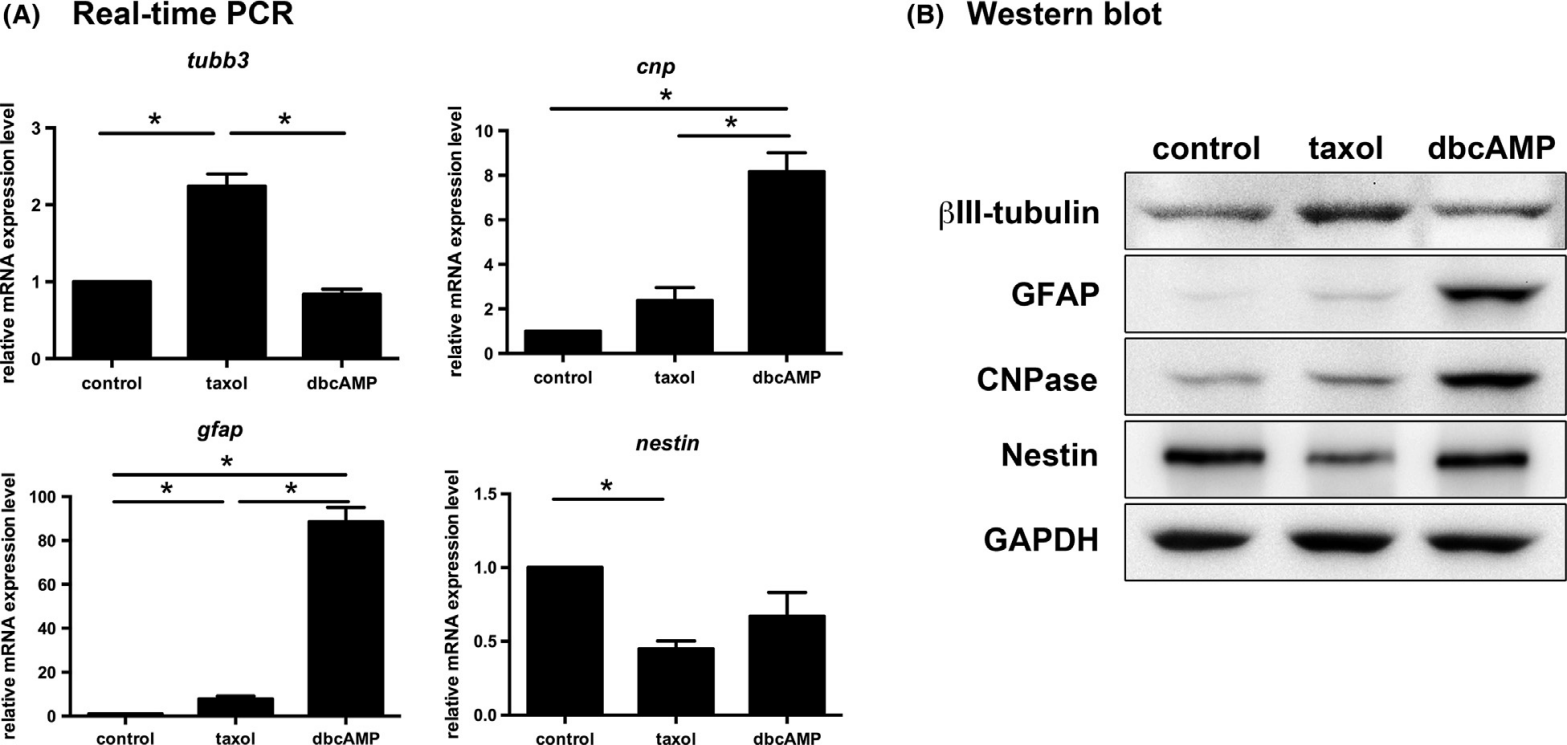
Characterizations of the mRNA and protein levels of *β* III‐tubulin, GFAP, CNPase, and nestin in taxol‐ and dbcAMP‐treated C6 cells by real‐time PCR and Western blot analysis. (A) mRNA levels of *tubb3*,* gfap,* and *cnp* were upregulated but *nestin* mRNA was downregulated in taxol‐treated C6 cells compared to the control group. The mRNA levels of target genes were calibrated based on the levels of *gapdh* mRNA. In dbcAMP‐treated C6 cells, mRNA level of *gfap*,* cnp,* and *nestin* were upregulated compared to control group. Data are presented as the mean ± SEM for three experiments in each group. *P < 0.01, unpaired Student's *t*‐test. (B) The immunoreactivities of *β* III‐tubulin, GFAP, CNPase proteins were upregulated while nestin was downregulated in taxol‐treated C6 cells. Nestin was downregulated in taxol‐treated C6 cells, while the immunoreactivities of *β* III‐tubulin were dramatically upregulated and the protein levels of GFAP and CNPase were also increased. In dbcAMP‐treated group, the immunoreactivities of GFAP and CNPase proteins were obviously increased but *β* III‐tubulin was not detectable compared to control group. The experiments were repeated three times and the results of a typical experiment are shown.

The neural differentiation patterns were examined by characterizations of the molecular markers for neural stem/progenitor cell (nestin), neurons (*β*III‐tubulin), astrocytes (GFAP), and oligodendrocytes (CNPase). The control C6 cells remained in an “undifferentiated” state as immunoreactive signals of nestin (Fig. 2A) was detected and immunoreactivity of the neural cell‐type‐specific markers *β*III‐tubulin, GFAP and CNPase was hardly detectable (Fig. 2B and C). After treatment with 100 nmol/L taxol for 48 h, immunoreactivities of GFAP, CNPase, and thick bundles of *β*III‐tubulin were found (Fig. 2D–F). Furthermore, the differentiation patterns caused by taxol and dbcAMP were compared as dbcAMP has been reported to induce astrocytic differentiation of C6 cells (Yoshimura et al. 1997; Takanaga et al. 2004). After dbcAMP treatment, the bipolar‐ and multiple‐processes extended from the cells (Fig. S1A). Immunofluorescent staining results confirmed that dbcAMP induced neural differentiation of C6 cells, as *β*III‐tubulin‐, GFAP‐, and CNPase‐positive cells appeared after dbcAMP treatment (Fig. S1B–D). mRNA and protein level of these neural differentiation markers in control and drug‐treated cells were examined by real‐time PCR and Western blotting (Fig. 3). The data showed that mRNA levels of the markers were consistent with protein levels in the cells after 48 h drug treatments. *β*III‐tubulin, GFAP, and CNPase were upregulated and nestin was downregulated in taxol‐treated C6 cells compared to the untreated C6 cells. After dbcAMP treatment, upregulated GFAP and CNPase were detected in C6 cells. These results suggested that neural differentiation occurred after taxol challenge in C6 cells.

### Increased percentage of neural differentiated C6 cells after taxol treatment

**Figure 4:**
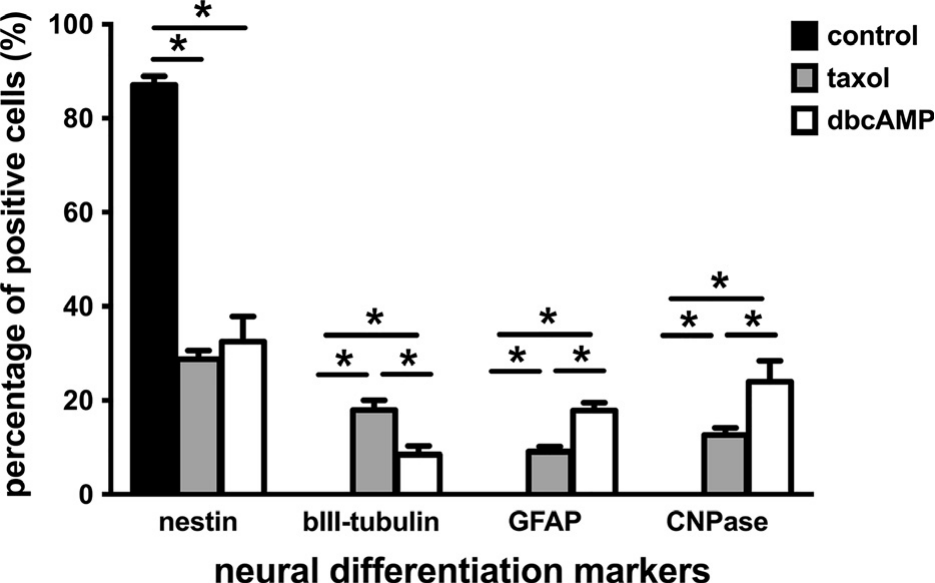
Percentages of nestin‐, *β* III‐tubulin‐, GFAP‐, and CNPase‐positive cells relative to the total number of taxol‐ and dbcAMP‐treated C6 cells. Neural cell‐specific molecular markers in control, taxol‐, and dbcAMP‐treated C6 cells were examined by immunocytochemical staining and then the immunostained cells were quantified. The percentages of nestin‐positive cells in taxol‐ and dbcAMP‐treated samples were significantly less than that in the control group. On the contrary, the percentages of neural differentiated cells in drug‐treated cells were significantly greater than that in the control groups. Data are shown as mean ± SEM for three experiments in each group. *P < 0.01, unpaired Student's *t*‐test.

The numbers of cells with various neural stem cell and differentiation markers existed in control experiment, taxol or dbcAMP treatment samples were quantified by image analysis for immunocytochemical staining. The percentages of nestin, *β*III‐tubulin, GFAP, and CNPase‐positive cells were 33%, 8%, 18%, and 24% in the dbcAMP‐treated C6 cells. Similarly, the percentages of nestin‐positive C6 cells decreased to 29% under taxol challenge. The percentages of *β*III‐tubulin‐, GFAP‐, and CNPase‐positive cells were increased to 18%, 9%, and 13%, respectively. The percentages of neural differentiated cells in control and drug‐treated groups were calculated and plotted statistically in Figure 4. Together with above experimental results, including morphological changes and positive signals of neural differentiation markers, we suggest that neural differentiation could be induced by taxol in C6 cells.

### Coexpression of *β*III‐tubulin and NFL was detected in taxol‐treated C6 cells

**Figure 5:**
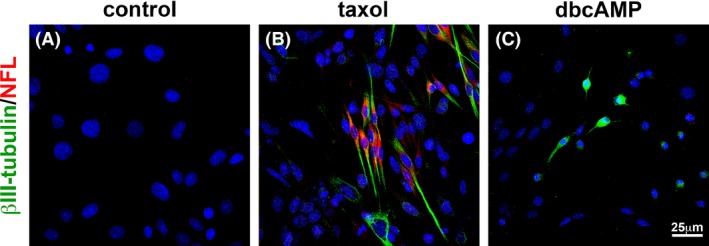
Coexpression of *β* III‐tubulin and NFL detected in taxol‐treated C6 cells by immunofluorescent staining. (Α) *β* III‐tubulin and NFL proteins were not detectable in control cells. (B) Coexpression of *β* III‐tubulin (green) and NFL (red) proteins was found in 100 nmol/L taxol‐treated C6 cells. (C) NFL was not detectable in dbcAMP‐treated cells. Cell nuclei are labeled in blue. Bar scale = 25 *μ*m.

The initial neurofilament protein NFL expression can be detected at the beginning of neuronal differentiation (Lariviere and Julien 2004). Thus, the expression of NFL under taxol and dbcAMP challenge was analyzed by immunofluorescence staining. In control group, immunoreactive signals of *β*III‐tubulin and NFL were undetectable (Fig. 5A). In 100 nmol/L taxol‐treated C6 cells, the immunoreactivities of neuronal markers *β*III‐tubulin and NFL were detected (Fig. 5B). As expected, dbcAMP induced differentiation of C6 cells into astrocytes with positive GFAP signals while NFL signal was difficult to detect (Fig. 5C). The results suggested that in comparison to astrocytic differentiation triggered by dbcAMP, taxol might induce neuronal differentiation in C6 glioma cells.

## Discussion

### Taxol may induce neural differentiation by elevating mRNA and protein levels of *β*III‐tubulin, GFAP, and CNPase

To clarify the neural differentiation process in cell or animal models, *β*III‐tubulin, GFAP, CNPase, and Nestin have often been examined for investigating the related mechanism (Caccamo et al. 1989; Svendsen et al. 2001). Recently, wogonin, a flavonoid anticancer drug isolated from *Scutellaria baicalensis* root, was found to trigger rat embryonic cortical neural precursor cells differentiation and neurite outgrowth (Lim et al. 2010). After combination treatment of indomethacin and IBMX, the proliferation of SCLC (small cell lung cancer cell) lines was inhibited and expression of NCAM and L1 (markers for neurons) increased without morphological changes (Lange et al. 2011). Taxol is one of the widely used antineoplastic drugs against many types of solid tumors. In our study, upregulated expressions of *β*III‐tubulin, GFAP, and CNPase were detected in taxol‐treated C6 cells. The data indicated that the chemotherapeutic effects of taxol may include elimination of the malignant cancer cells as well as induction of differentiation of the glioma cells into neurons, astrocytes, and oligodendrocytes. Thus, when the tumor cells are driven toward terminal differentiation, the proliferative potential of the cancer cells will be lost. An example of this effect is that taxol inhibits tumor growth by inducing ovarian cancer epithelial cells toward a benign fibroblast‐like phenotype (Jia et al. 2012).

### Taxol increases mRNA and protein levels of *β*III‐tubulin in C6 cells

The antitumor activity of taxol is through a unique mechanism by which taxol blocks cell cycle in its G1 or M phase by stoichiometrically binding to microtubules and hyperstabilizing the structure (Xiao et al. 2006). We found that inhibition to C6 cell growth by taxol was dose‐dependent by performing MTT cell viability assay. Similar results of concentration‐dependent effects have been observed in other cancer cells, for example, breast and lung cancer cell lines (Das et al. 2001; Pushkarev et al. 2004; Wu et al. 2007). In addition to the occurrence of cell death, morphology changes were shown in taxol‐treated C6 cells, including cell elongation, cellular processes thinning, irregular shapes, and fragmented nucleation or micronuclei. These cellular changes might result from taxol stabilization of microtubules, which are important in maintenance of cell shape and cell motility (Hari et al. 2003; Lee et al. 2007; Umezu et al. 2008; Kavallaris 2010). In our study, the expression of *β*III‐tubulin was upregulated in taxol‐treated C6 cells as detected by real‐time PCR, Western blot, and immunocytochemistry. Similar results were reported previously (Ranganathan et al. 1998; Katsetos et al. 2007). In human prostate cancer cell line DU‐145, increased mRNA and protein expression of *β*III‐tubulin were found after taxol treatment (Ranganathan et al. 1998). The treatment of human glioblastoma cell line T98G exposed to taxol and the treatment led to the formation of *β*III‐tubulin composed microtubule bundles (Katsetos et al. 2007). It has been reported that *tubb3* is a factor of taxol resistance in cancer, such as lung cancer cells could be sensitized to taxol by downregulating *tubb3* expression with antisense oligonucleotides (Kavallaris et al. 1999). In addition to taxol resistance, *β*III‐tubulin plays an important role in neural differentiation (Katsetos et al. 2003). Thus, we also examined another neuronal marker NFL for characterization the differentiation pattern of taxol‐treated C6 cells. We found that *β*III‐tubulin and NFL coexisted in the taxol‐treated neuronal‐like C6 cells. These results indicate that the *β*III‐tubulin level increases in response to taxol challenge. Drug‐induced neuronal differentiation may provide a solution for the cells to conquer the suppression of microtubule dynamics caused by taxol.

### Taxol‐induced redistribution of intermediate filaments may be involved in astrocytic differentiation

In our studies, immunocytochemical staining revealed that the percentages of GFAP‐ and CNPase‐positive cells in the dbcAMP‐treated C6 cells were higher than that of *β*III‐tubulin‐positive cells. Astrocytes having round cell bodies and thin processes appeared after dbcAMP treatment and could be recognized by anti‐GFAP antibody as reported previously (Yoshimura et al. 1997; Takanaga et al. 2004). Moreover, increased CNPase enzymatic activity has been detected by dbcAMP administration in C6 cells and rat oligodendrocytes (McMorris 1977, 1983). To summarize, neural differentiation was induced by dbcAMP administration in C6 cells, especially astrocyte and oligodendrocyte differentiation. When treating C6 cells with taxol, the percentages of GFAP‐ and CNPase‐positive cells were 9% and 13%, respectively. It has been shown that redistribution of GFAP in cortical astrocyte culture occurs following taxol treatment and the phenomenon indicates some microtubule‐targeting drugs could cause dramatic changes in the distribution of intermediate filaments and, as a consequence, in the astroglial shape (Goetschy et al. 1986). The mechanisms of neural differentiation induced by taxol still need to be further clarified. Here, we suggest that taxol‐induced C6 cell shape alteration could be due to redistributing the intermediate filaments and it might be involved in neural differentiation.

## Conflict of Interest

We certify that there are no known conflicts of interest associated with this publication and there has been no significant financial support for this work that could have influenced its outcome.

## Supporting information


**Figure S1.** The expression of neural differentiation markers induced by dbcAMP in C6 cells as demonstrated by immunofluorescent staining.
**Figure S2.** Examination of cell viability and neural differentiation markers induced by taxol or dbcAMP in C6 cells.
