# Supplementary material for: Induction of neural differentiation in rat C6 glioma cells with taxol
Source: Brain Behav. 2015 Oct 26;5(12):e00414. doi: 10.1002/brb3.414 (PMC4667627; doi:10.1002/brb3.414)

**Supplementary Figure 1. The expression of neural differentiation markers induced by dbcAMP in C6 cells as demonstrated by immunofluorescent staining**

C6 cells were cultured in adhesion condition and treated with 100nM dbcAMP for 2 days. The morphological images were recorded by phase contrast microscopy (A). Bar scale=100 µm. βIII-tubulin-, GFAP- and CNP-positive cells were identified after dbcAMP treatment (B, C and D). Bar scale=25 µm.


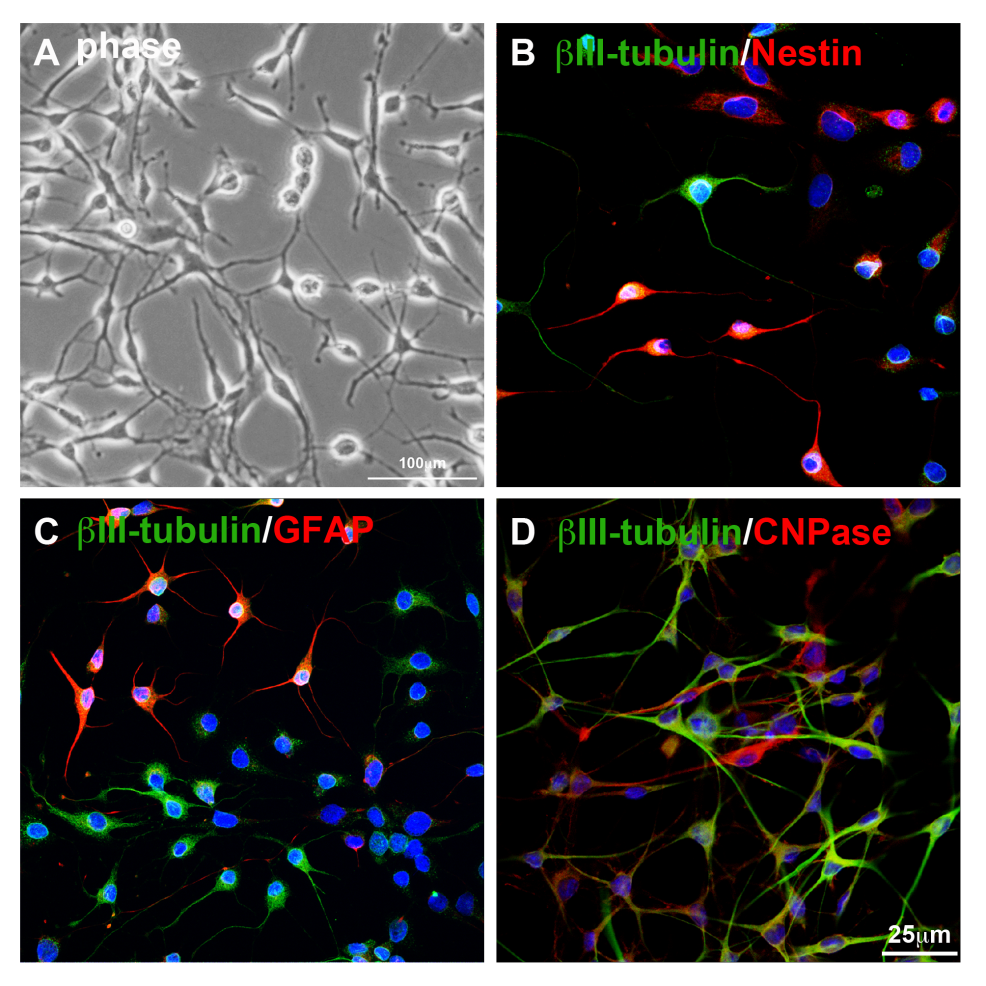


**Supplementary Figure 2. Examination of cell viability and neural differentiation markers induced by taxol or dbcAMP in C6 cells**

C6 cells were plated at a density of 1000 cells/96-well plates or 1 × 105 cells/6-cm dish. 5 different drug treatments were designed to confirm if the differentiation was reversible. After 24hr incubation, cells were exposed to medium containing taxol or dbcAMP for 2 days or 4 days. After 2-day drug treatment, the medium was removed and replaced by complete medium. Cells with no drug treatment served as a control group. At the end of treatment, cells were collected for cell viability assay and Western blot analysis. After taxol treatment, cell viability decreased to 75% and 70% on day 2 and day 4 respectively. When the medium containing taxol was replaced with complete medium, the cell viability was recovered (80% on day 1 and 90% on day 2). Similar results were observed in ibmx-treated cells. The immunoreactivities of phospho-STAT3 and GFAP could be induced by dbcAMP, while the immunoproduct disappeared when dbcAMP was removed. This result indicated the dbcAMP-induced astrocytic differentiation could be reversible. phosphor-mTOR was chosen as an indicator for testing taxol-induced differentiation. Upregulation of phosphor-mTOR and βIII-tubulin was shown after taxol stimulation by Western blot analysis. 2 days after taxol was removed for the culture medium, phospho-mTOR and βIII-tubulin remained high expression level. The results showed irreversible differentiation may happen after taxol challenge.


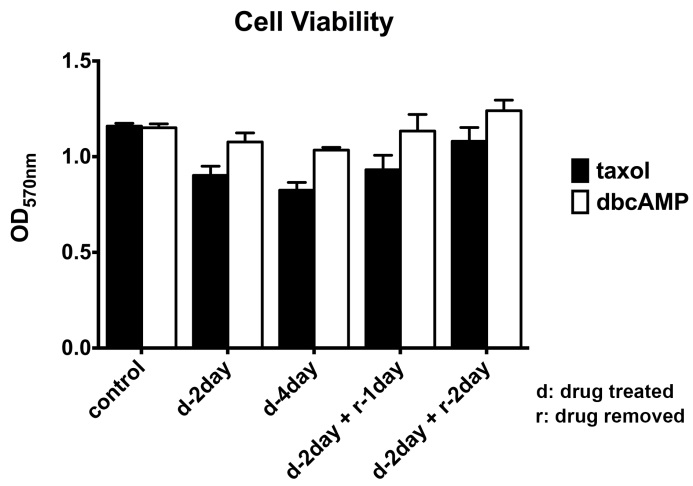


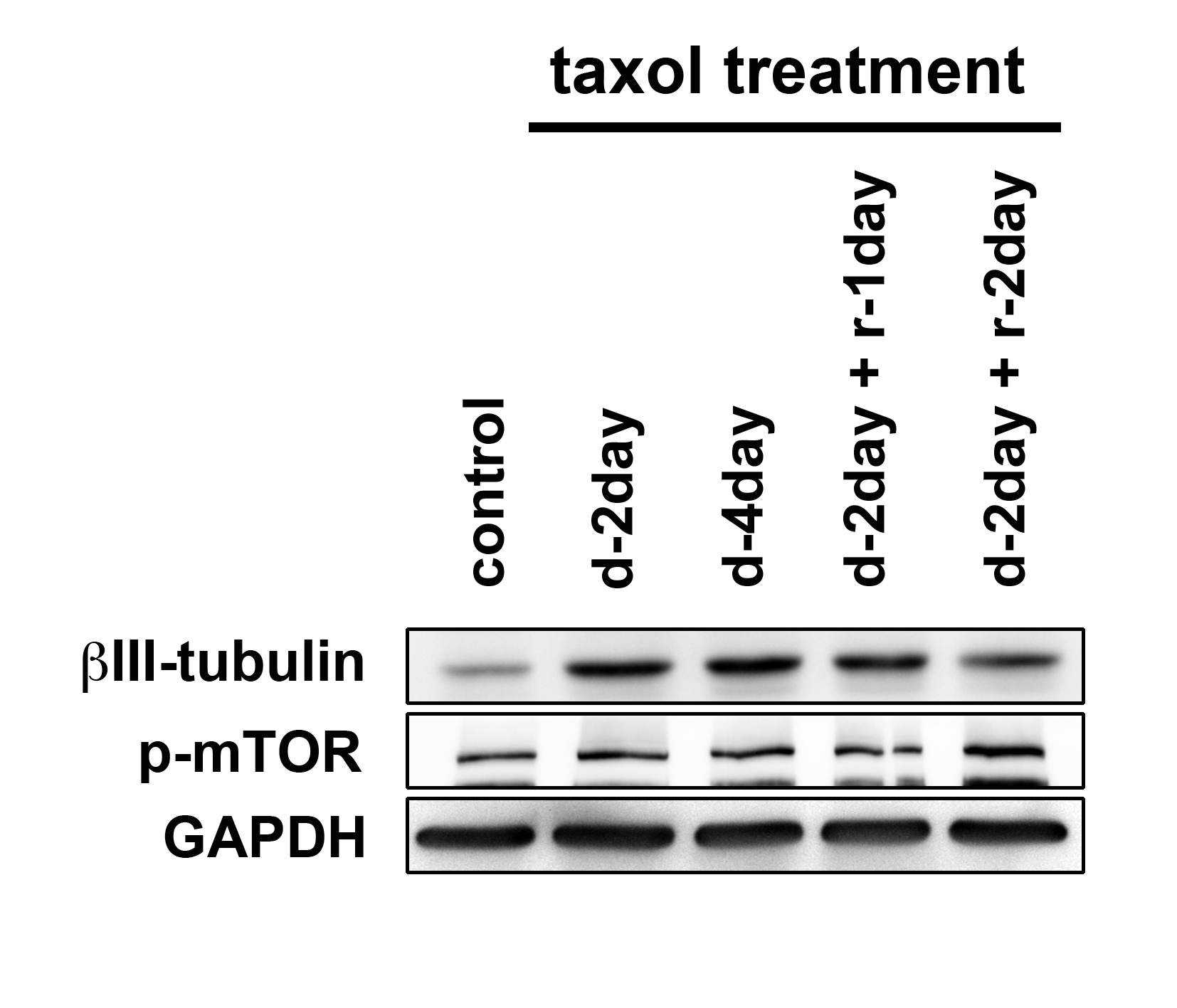

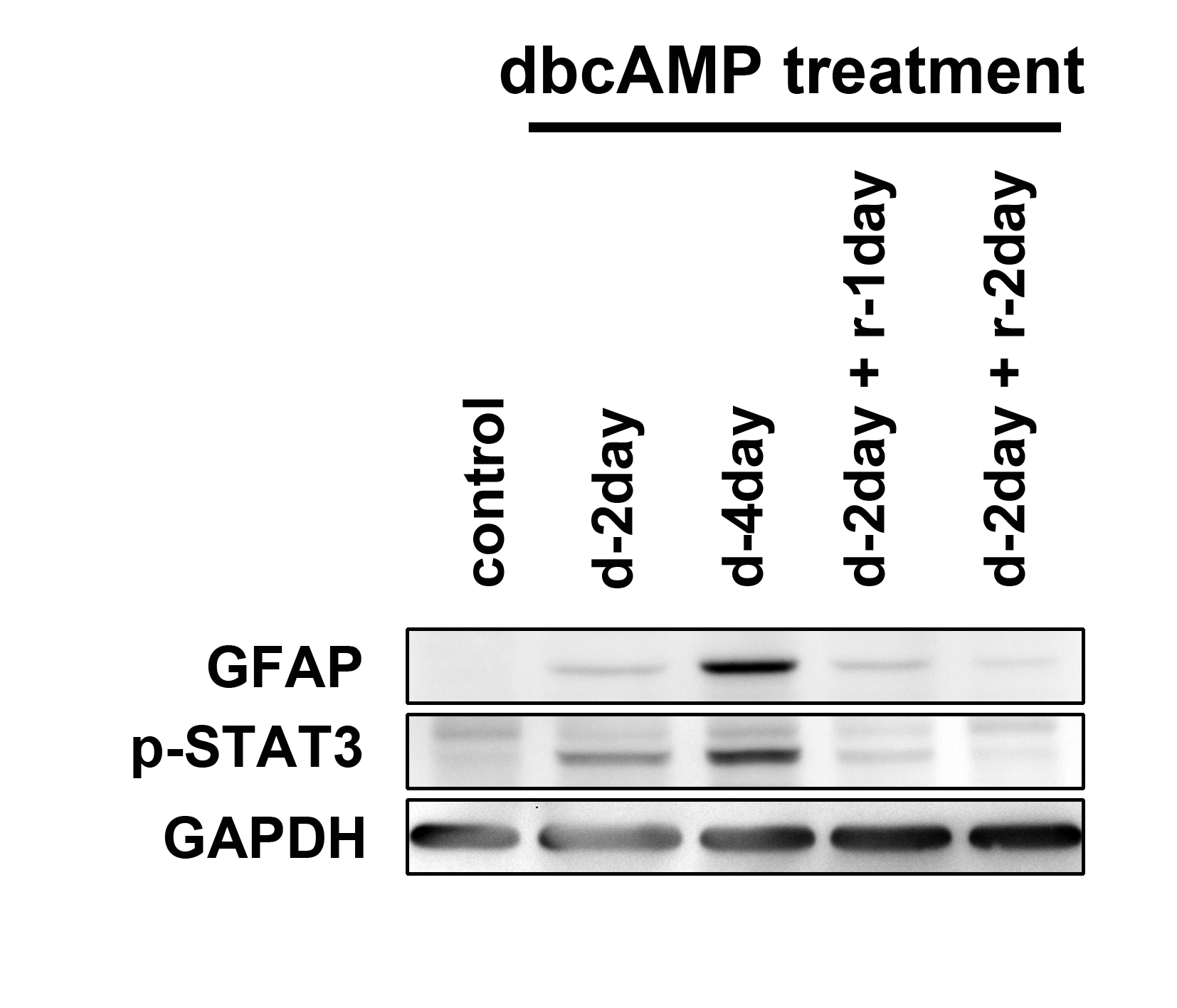

Supplement: Supplementary file 1 — Figure S1. The expression of neural differentiation markers induced by dbcAMP in C6 cells as demonstrated by immunofluorescent staining. Figure S2. Examination of cell viability and neural differentiation markers induced by taxol or dbcAMP in C6 cells. [file BRB3-5-e00414-s001.docx]
